# 1248. Implementing Infection Prevention and Control with Antimicrobial Stewardship in a Tertiary Care Hospital in India: An Interprofessional Education and Collaborative Practice Approach

**DOI:** 10.1093/ofid/ofad500.1088

**Published:** 2023-11-27

**Authors:** K Dr Raksha, Kukkamalla Anand, Peralam Yegneswaran Prakash

**Affiliations:** St. Martha's Hospital, Bangalore, Karnataka, India; Manipal University College Malaysia, Manipal, Karnataka, India; Kasturba Medical College, Manipal, Manipal, Karnataka, India

## Abstract

**Background:**

Infection Prevention & Control (IPC) along with Antimicrobial Resistance (AMR) has complex problems, it requires distinctive disciplinary efforts. There are few data concerning the facilitators and barriers in India. Interprofessional practice and education (IPE & IPP) initiatives amongst various stakeholders is one amongst them. Current approaches to IPC & AMR are fragmented and unidisciplinary, more like ''An Elephant and the Blind Men'. Hence the need of this structured study.

**Methods:**

We conducted a prospective interventional study for one year with an interprofessional team. After institutional ethical committee clearance, an Antimicrobial Stewardship team was formed which devised training modules validated by Delphi technique /& ADDIE (Analyze, Design, Develop, Implement & Evaluate) approach by the Infection Control Committee and external resource persons. Simulated sessions, case based discussions, patient education videos & pamphlets, online interactive classrooms, ward & laboratory rounds were instituted for all doctors, staff nurses, laboratory technicians, pharmacists, and patients. Key quality indicators for antimicrobials prescription & consumption was defined and monitored. High end antimicrobial policy was implemented. Checklists & surveillance forms were put in use. Feedback surveys and audits were conducted to assess the effectiveness of these interventions.

**Results:**

We observed a statistically significant increase in compliance to hand hygiene, health care associated infections (HAI) monitoring, reporting of needle stick injury, adherence to isolation protocols, and antibiotic policy (P value< 0.001) across all units. Maximum compliance to antibiotic policy was observed in medical intensive care units, least in nephrology & neonatal intensive care. Pre-test & post-test questionnaires were used for assessment. An improvement of ∼78 % in the knowledge domain was seen after the sessions. Key quality indicators for HAIs showed statistically significant improvement (P value< 0.001).

**Conclusion:**

A multimodal interprofessional approach aids the training aspect of IPC & AMSP. Such structured programs in the curriculum addresses the silent pandemic of health care associated infections and antimicrobial resistance.

**Disclosures:**

**All Authors**: No reported disclosures

